# Nerve transfer for restoration of lower motor neuron-lesioned bladder, urethral, and anal sphincter function in a dog model. Part 3. nicotinic receptor characterization

**DOI:** 10.1152/ajpregu.00273.2022

**Published:** 2023-07-17

**Authors:** Nagat Frara, Mary F. Barbe, Dania Giaddui, Danielle S. Porreca, Alan S. Braverman, Ekta Tiwari, Attia Ahmad, Justin M. Brown, Benjamin R. Johnston, Stanley F. Bazarek, Michael R. Ruggieri

**Affiliations:** ^1^Center for Translational Medicine at the Lewis Katz School of Medicine, Temple University, Philadelphia, Pennsylvania, United States; ^2^Department of Neurology, Thomas Jefferson University Hospital, Philadelphia, Pennsylvania, United States; ^3^School of Engineering, Brown University, Providence, Rhode Island, United States; ^4^Cooper Medical School of Rowan University, Camden, New Jersey, United States; ^5^Department of Neurosurgery, Massachusetts General Hospital, Boston, Massachusetts, United States; ^6^Department of Neurosurgery, Brigham & Women’s Hospital, Boston, Massachusetts, United States

**Keywords:** epibatidine-induced contractions, muscarinic receptor, neuromuscular, nicotinic receptor subunit, sodium channel

## Abstract

Very little is known about the physiological role of nicotinic receptors in canine bladders, although functional nicotinic receptors have been reported in bladders of many species. Utilizing in vitro methods, we evaluated nicotinic receptors mediating bladder function in dogs: control (9 female and 11 male normal controls, 5 sham operated), Decentralized (9 females, decentralized 6–21 mo), and obturator-to-pelvic nerve transfer reinnervated (ObNT-Reinn; 9 females; decentralized 9–13 mo, then reinnervated with 8–12 mo recovery). Muscle strips were collected, mucosa-denuded, and mounted in muscle baths before incubation with neurotransmitter antagonists, and contractions to the nicotinic receptor agonist epibatidine were determined. Strip response to epibatidine, expressed as percent potassium chloride, was similar (∼35% in controls, 30% in Decentralized, and 24% in ObNT-Reinn). Differentially, epibatidine responses in Decentralized and ObNT-Reinn bladder strips were lower than controls after tetrodotoxin (TTX, a sodium channel blocker that inhibits axonal action potentials). Yet, in all groups, epibatidine-induced strip contractions were similarly inhibited by mecamylamine and hexamethonium (ganglionic nicotinic receptor antagonists), SR 16584 (α3β4 neuronal nicotinic receptor antagonist), atracurium and tubocurarine (neuromuscular nicotinic receptor antagonists), and atropine (muscarinic receptor antagonist), indicating that nicotinic receptors (particularly α3β4 subtypes), neuromuscular and muscarinic receptors play roles in bladder contractility. In control bladder strips, since tetrodotoxin did not inhibit epibatidine contractions, nicotinic receptors are likely located on nerve terminals. The tetrodotoxin inhibition of epibatidine-induced contractions in Decentralized and ObNT-Reinn suggests a relocation of nicotinic receptors from nerve terminals to more distant axonal sites, perhaps as a compensatory mechanism to recover bladder function.

## INTRODUCTION

Bladder function is controlled by multiple receptor-signaling pathways taking part in bladder emptying (1). Released neurotransmitter acetylcholine (ACh) interacts with a variety of cholinergic receptor subtypes and causes bladder smooth muscle contractions. Among these are muscarinic receptors (mAChRs) that play a significant role in controlling bladder contractions (2, 3). Other cholinergic receptors include nicotinic receptors (nAChRs), which are ion channels that open upon binding of ACh to initiate influx of Na^+^ and Ca^2+^ ions (4). Although the role of mAChRs in bladder contraction is well studied, the importance of nAChRs in modulation of bladder contractility is still incompletely understood.

In mouse bladders, neuronal nAChRs necessary for bladder function have been found in parasympathetic intramural ganglia (5, 6), and nAChR activation induces detrusor muscle contractions by facilitating fast synaptic transmissions between preganglionic and postganglionic neurons (7). This ganglionic nAChR subtype is made up of α3 and β4 subunits (7). In rat bladders, functional nAChRs have been found in the urothelium, nerve terminals, and on nerve fibers located within muscle layers (8, 9), as well as on spinal cord neurons and postsynaptic neurons in pelvic ganglia (10). The Masuda study found no functional nAChRs in the detrusor muscle layers (10), although a different study indicated that bladder contractions are mediated through nAChRs located on postsynaptic parasympathetic nerve fibers in detrusor muscle, as well as in pelvic ganglia neurons (11). Guinea pig and rabbit bladder strip findings are similar, showing that nAChRs responsible for induced ACh release and strip contractions are limited to neuronal components (autonomic ganglion cells and parasympathetic cholinergic nerve terminals), with no evidence of these receptors in detrusor muscle layers (12–15). A study in dog and cat bladders reported the presence of ganglionic receptors in detrusor muscle layers, but no evidence of their presence on pre- or postganglionic parasympathetic nerve terminals (16). In humans, nAChRs have been found on urothelial cells (17), and neuronal nAChR subtypes on peripheral nerves (18). Collectively, these studies indicate that there might be multiple sites of action and/or receptor subtypes, as well as differences between species and experimental paradigms that account for the effects of nAChRs on the bladder.

Therefore, we sought here to gain further insight on the role of nAChRs in dog bladder function by studying their pharmacological properties in the urinary bladders of first normal control dogs, as well as in long-term decentralized dogs, and long-term decentralized dogs that underwent reinnervation. Dogs were chosen because they have physiological similarities to humans and are close in anatomical scale. Dogs are also suitable for clinical measurements, neurophysiological studies, and pharmacological investigations. This study is an add-on investigation to a long-term project designed to develop novel surgical approaches for restoration of bladder, urethral, and anal sphincter control to lower motoneuron-lesioned pelvic organs. Specifically, in a dog model, we performed long-term extensive decentralization and nerve rerouting surgeries following long-term decentralization with the ultimate goal of restoring bladder function. Using bladder smooth muscle strips from these studies, we reported that smooth muscle contractile properties were preserved postdecentralization, suggesting that mAChRs taking part in bladder contractions are still functional after long-term bladder decentralization (19, 20).

Here, utilizing in vitro smooth muscle strip neuropharmacological methods, we extended these past studies to now evaluate the response to cholinergic receptors, including nicotinic, that might mediate bladder function in control dogs, long-term decentralized dogs, and long-term decentralized and then reinnervated dogs, and whether this decentralization and reinnervation procedure alters receptor responses (or location) to different agonists and antagonists. Expanded information on receptors responsible for bladder activity in these surgical models may help in finding suitable drugs for maintaining and enhancing functional recovery after bladder denervation.

## MATERIALS AND METHODS

### Animals

All studies were approved by the Institutional Animal Care and Use Committee according to guidelines of the National Institute of Health for the Care and Use of Laboratory Animals and the United States Department of Agriculture and the Association for Assessment and Accreditation of Laboratory Animal Care (Animal Care and Use Protocol No. 5043). This study utilized a total of 43 male and female dogs, including 25 controls (9 normal control females, 11 normal control males, 5 sham-operated control females), 9 decentralized females, and 9 reinnervated females. Forty-two were mixed-breed hound dogs (31 females and 11 males), 6–8 mo old, weighing 20–25 kg (Marshall BioResources, North Rose, NY). The remaining female dog was an adult beagle, 8 mo old, that was obtained from Envigo Global Services, Inc., Denver, PA. Dogs were group housed according to the institution’s standard husbandry with 12-h exposure to light/dark cycles. In the control group, 13 of 14 females and all 11 males were unoperated or sham-operated control animals derived from other larger studies focusing on nerve transfer for pelvic organ reinnervation or heart failure.

### Surgical Decentralization of the Bladder

Decentralization surgeries were performed on 18 animals, as previously described (19). Briefly, animals were fasted the day before surgery and received 20 mg/kg iv cefazolin with redosing every 4 h until procedure completion. Postoperative antibiotic prophylaxis included 30 mg/kg cephalexin, twice a day for up to 5 days. Animals were sedated using propofol (6 mg/kg iv) for endotracheal intubation, and then anesthesia maintained using isoflurane (2%–4% mean alveolar concentration) in oxygen. Both urethral and bladder pressures were monitored by dual balloon catheters in the bladder. Decentralized and obturator-to-pelvic nerve transfer reinnervated (ObNT-Reinn) animals (*n* = 9/group) were subjected to laminectomy of the L6 through sacral (S)2 vertebrae to expose the lower spinal cord and to identify sacral spinal roots. Decentralization was achieved by bilateral transection of L7 dorsal roots and all dorsal and ventral roots caudal to L7. Spinal ganglia were completely removed in five Decentralized and six ObNT-Reinn animals yet remained intact with their connections to the spinal cord removed in four Decentralized and three ObNT-Reinn animals. Hypogastric nerves were accessed in the abdomen and bilaterally transected. Ten- to 15-mm sections were removed from each transected root or nerve for complete separation. At the conclusion of surgery, all Decentralized and ObNT-Reinn animals underwent tail amputation as a protection from self-mutilation that was found to occur as a result of the decentralization. Animals in the Decentralized-only group were provided 6–21 mo of postoperative recovery before euthanization and bladder tissue collection, whereas ObNT-Reinn animals that were decentralized were provided 9–13 mo of postoperative recovery before reinnervation.

Five sham-operated female control dogs underwent lumbosacral laminectomy and nerve root identification via electrical stimulation but no spinal root transection. These sham dogs also underwent abdominal opening for identification of hypogastric nerves, without transection of these nerves. These sham-operated dogs were provided 6–21 mo of postoperative recovery before euthanization and bladder tissue collection.

### Nerve Transfer Reinnervation Surgery

For the nerve transfer surgery, nine of the previously decentralized animals were anesthetized and catheterized with balloon catheters. Obturator nerves were accessed via abdominal surgery, identified, and divided longitudinally using a microscalpel. Approximately 75% of the obturator nerve was left intact to retain innervation of hind limb adductor muscles. The other quarter of the fascicles were transected, transferred, and sutured end-to-end to the transected anterior vesical branch of the pelvic nerve, bilaterally, using described methods (21, 22). Axoguard nerve connectors (Axogen Corp, Alachua, FL) were used to maintain transferred nerve coaptation and to reinforce the coaptation site that was covered with Tisseel fibrin sealant (Baxter, Deerfield, IL). These animals constitute the ObNT-Reinn group. An average 10-mo reinnervation recovery time (8–12 mo range) before euthanization and tissue collection was chosen based on data from the pilot study of this series that showed functional recovery of squat and void postures between 4 and 6 mo after obturator nerve transfer in three ObNT-Reinn animals (21). The reinnervated animals also underwent a pudendal nerve transfer for reinnervation of the anal sphincter, results of which have been partially reported (21).

### Postoperative Care

For each surgical procedure, buprenorphine (0.03–0.05 mg/kg) was administered subcutaneously, twice a day for 2 days postoperatively. Since the Decentralized and ObNT-Reinn animals were incontinent of urine because of the sacral root transection and loss of both pelvic and pudendal nerve function, the Crede’ maneuver was performed on all decentralized animals twice daily. The frequency of squat-and-void postures was recorded for 24 h at monthly intervals pre- and postoperatively, as previously reported (21).

Urinalysis results have been previously reported (19, 21). Although, all Decentralized and ObNT-Reinn animals had multiple instances of culture-confirmed bacteriuria, no catheterized urine specimens from animals collected before any surgery were culture positive (19, 21).

### In Vitro Muscle Strip Contractility

At study end, animals were deeply anesthetized as described earlier and whole bladders were harvested from five sham-operated controls, nine Decentralized, and nine ObNT-Reinn animals. Immediately thereafter, animals were euthanized by a terminal dose of Euthasol (pentobarbital sodium 86 mg/kg and phenytoin sodium 11 mg/kg iv). In addition, bladders were obtained from 11 normal male and nine normal female dogs used for other studies. Bladder tissues obtained from all five sham-operated, three of the normal control females, four of the Decentralized, and eight of the ObNT-Reinn were also used in a previous publication in which purinergic-induced responses were examined (19). We first examined for differences between the five sham-operated females and nine normal control females. No differences were observed, similar to our previous report of no differences between sham-operated and unoperated normal control female dogs. Therefore, these animals were combined into one group of 14 female controls. We next examined for differences between these 14 females and the 11 males; no differences were seen. Therefore, all control animals were combined into one group of 25 controls.

After collection, bladders were washed in Tyrode buffer, immersed in Custodiol HTK organ transport media, and saved on ice at 4°C (23). Using sharp micro scissors and ×5 magnifying loops, smooth muscle strips (mucosa denuded) were dissected from the body of the bladder at least 1 cm above the ureteral orifices, as previously described (23). Each strip was mounted between force transducers and positioners with a pair of spring wire clips (158802, Radnoti LLC, Covina, CA), in muscle baths containing 10 mL of Tyrode solution that were maintained aerated with 95% O_2_ and 5% CO_2_ at 37°C. Strips were initially stretched slowly to 20 milli Newtons (mN) of isometric tension and allowed to relax to ∼10 mN of basal tension (19, 23, 24). Contractile responses were monitored with isometric force transducers. Electrical field stimulation (EFS) of 12 V (V), 1 ms (ms) pulse duration, and 30 Hertz (Hz) frequency was delivered to each strip using a Grass S88 stimulator (Natus Neurology, Inc., Warwick, RI) interfaced with a Stimu-Splitter II (Med-Lab Instruments, Loveland, CO) power amplifier and LabChart software (ADInstruments). The maximal EFS-evoked response is reported. After equilibration for 30 min, bladder smooth muscle strips were induced to contract with an isotonic buffer containing 120 mM potassium chloride (KCl), and maximal responses were determined. After being washed and reequilibrated, subsets of strips were incubated with a selection of antagonists and agonists for ∼20 min. Then, epibatidine, a potent nAChR agonist (No. 0684, Tocris Bioscience, R&D Systems, Minneapolis, MN) was added at a final concentration of 10 μM, and maximal responses were measured. Epibatidine was chosen because it has a very high potency for nAChRs (∼120 times more potent than nicotine itself; 25). The 10 μM concentration of epibatidine used was as previously established in our laboratory (23), and according to the concentration-response curve generated after application of different concentrations of epibatidine (Supplemental Fig. S1*A*). Concentration response curves for SR 16584 and tubocurarine are provided in Supplemental Fig. S1, *B* and *C*. Concentrations of the other drugs were chosen within the effective concentration range typically used to selectively block the targeted nAChR subtype in vitro (26–31). Before the end of each experiment, strips were treated with 30 μM of the mAChR agonist bethanechol to validate that the muscle strips are still viable after drug treatments and to determine that drugs used in this study, including epibatidine, do not interact directly with the mAChR by causing either an activation or inhibition of the bethanechol-induced strip contractions. Also, before bethanechol treatment, EFS of 12 V, 1 ms pulse duration, and 30 Hz frequency was delivered to each strip to assess strips’ responses to neural stimulation after different drug treatments. For example, it has been reported that a drug could be acting on prejunctional receptors if it alters responses to EFS with no effect on the response to myogenic stimulation. However, if a drug alters responses to both electric field and myogenic stimulations, it could be acting on postjunctional or both pre- and postjunctional receptors (32).

Only responses to KCl and EFS are presented in milliNewtons. The other data have been normalized to percentage of the KCl response. As explained early in the results, responses to KCl or epibatidine did not differ between males and females in the control group (Supplemental Fig. S2, *A* and *B*). Therefore, these data were combined. Also, as described previously by our group (23), a strip’s force of contractions was not normalized to strip size because each strip was mounted along its length between two spring wire clips. Thus, the amount of muscle involved in generating the force is very similar for all strips regardless of strip size. Use of this methodology also matches previously reported methods from our laboratory (19, 33, 34).

### Agonists and Antagonists Used

Atropine sulfate monohydrate salt (No. A0257), tetrodotoxin (TTX, No. 554412), tubocurarine chloride hydrate (No. T2379), mecamylamine hydrochloride (No. M9020), methyllycaconitine citrate hydrate (MLA, No. M168), hexamethonium bromide (hexane-1,6-bis[trimethylammonium bromide], No. H0879), 1,1-dimethyl-4-phenyl-piperazinium iodide (DMPP, No. D5891), and bethanechol chloride (No. 1071009) were obtained from Sigma-Aldrich, St. Louis, MO. α,β-Methylene adenosine triphosphate (α,β-mATP; α,β-methylene adenosine 5′-triphosphate trisodium salt, No. 3209), A-803467 [5-(4-chlorophenyl)-*N*-(3,5-dimethoxyphenyl)-2-furancarboxamide, No. 2976], dihydro-β-erythroidine hydrobromide (DHβE, No. 2349), SR 16584 {1,3-dihydro-1-(3-exo)-9-methyl-9-azabicyclo[3.3.1]non-3-yl]-2*H*-indol-2-one, No. 4424}, and α-conotoxin AuIB (No. 3120) were obtained from Tocris Bioscience, R&D Systems, Minneapolis, MN. Atracurium besylate was obtained from LKT Laboratories, Inc. (No. A7668, St. Paul, MN). For stock solutions (0.03–100 µM), all drugs were dissolved in distilled water, except for A-803467 (dissolved in ethanol) and SR 16584 (dissolved in dimethyl sulfoxide).

Because of the progression of this study over time, in vitro contractility to the various drugs used were tested only in a subset of each group’s animals, per individual experiment, with the exact numbers of animals per group reported in each figure as *N*. We calculated the average number of animals per treatment and average number of strips per treatment. Presented as means ± 95% CI, the number of control animals was 7.2 ± 2.1 per treatment, with 102.3 ± 83.5 strips per treatment; the number of Decentralized animals was 4.4 ± 0.7 per treatment, with 54 ± 41.8 strips per treatment; and the number of ObNT-Reinn subjects was 5.9 ± 0.8 per treatment, with 58.9 ± 42.9 strips per treatment. The numbers of animal specimens per group and per treatment (indicated as *N*), and muscle strips per treatment (indicated as *n*), are listed in the figures.

### Statistical Analyses

Statistical analyses were performed using Prism 8.4.2 or 9 (GraphPad Software, La Jolla, CA). Muscle strip data are presented as medians [with their interquartile ranges (IQR)]. For all data and figures, we first calculated the mean of multiple strips tested per animal and then used the mean of each animal in the statistical analyses. Data were analyzed using nonparametric tests. When there were only two independent groups, a Mann–Whitney test was used (unpaired and two-tailed). When there were more than two groups, a Kruskal–Wallis ANOVA test was used, followed by Dunn’s multiple comparisons post hoc tests. *P* values were adjusted for multiple comparisons whenever applicable and values of 0.05 or less were considered statistically significant for all analyses. The reported error bars and *P* values are based on the animals (with each animal being the mean of all strips), so data analyses were based on biological replicates (a dog), not on technical replicates (a strip; 35).

This study is exploratory and did not test a prespecified statistical null hypothesis (36); therefore, the calculated *P* values are interpreted as descriptive, not hypothesis testing.

## RESULTS

### No Sex Differences Are Seen between Detrusor Muscle Strip Responses to KCl or Epibatidine

We first examined differences in strip responses between males and females in control bladders to 120 mM KCl, a cell membrane depolarizer, or to 10 µM epibatidine, a nAChR agonist. Smooth muscle strip responses to KCl and epibatidine were similar in bladders from control males versus females (Supplemental Fig. S2, *A* and *B*). Thus, data from controls of both sexes were pooled for all further analyses.

### Strip Responses to KCl or Epibatidine Are Not Different between Surgical Groups

Smooth muscle strip responses to KCl were similar between the three groups (Supplemental Fig. S3, Table 1), as were their responses to epibatidine (Supplemental Fig. S4, Table 1). The average strip response to epibatidine, measured as percentage (%) of KCl-induced contraction, was ∼35% in control bladder strips, 30% in Decentralized (∼14% less than the control response), and 24% in ObNT-Reinn (∼32% and 20% less than control and Decentralized bladders, respectively). There were no statistically significant differences between groups. This data suggests that functional nAChRs are present even after bladder decentralization, or decentralization and then reinnervation.

**Table 1.** Summary of treatment responses in muscle strips from control, Decentralized, and obturator nerve transfer reinnervated bladders

Agonist/Antagonist Treatment	Primary Action	Control	Decentralized	ObNT-Reinn	Control vs. Decentralized	Control vs. ObNT-Reinn	Decentralized vs. ObNT-Reinn
		*mN*					
KCl (120 mM), Supplemental Fig. S3	Muscle cell membrane depolarization, inducing contraction	↑ 19 (15–22)	↑ 21 (14–25)	↑ 22 (17–32)	n.s.	n.s.	n.s.
		Epibatidine-induced contractions (%KCl)					
Epibatidine (10 µM), Supplemental Fig. S4	nAChR agonist (potent, yet nonselective for nAChR subtypes)	↑ (35%)	↑ (30%)	↑ (24%)	n.s.	n.s.	n.s.
TTX (1 µM), Fig. 1*A*	Na^+^ channel blocker (inhibits nerve action potentials)	No effect	↓ (49%)	↓ (47%)	*P = 0.01	*P = 0.02	n.s.
A-803467 (1 µM), Fig. 1*A*	Selective Na^+^ channel Nav1.8 blocker (TTX resistant)	No effect	No effect	No effect	n.s.	n.s.	n.s.
Hexamethonium, (100 µM), Fig. 2*A*	Ganglionic nAChR antagonist; blocks α3 subunit	↓ (99%)	↓ (99%)	↓ (100%)	n.s.	n.s.	n.s.
Mecamylamine, (10 µM), Fig. 2*B*	Ganglionic nAChR antagonist (at low concentrations >> muscle nAChR (at high concentrations)	↓ (97%)	↓ (87%)	↓ (97%)	n.s.	n.s.	n.s.
DMPP (100 µM, %KCl), Supplemental Fig. S5	Ganglionic nAChR agonist (nonselective; blocks transmission of preganglionic impulses)	↑ (5%)	↑ (7%)	↑ (7%)	n.s.	n.s.	n.s.
SR 16584, Fig. 3	Ganglionic nAChR α3β4 selective antagonist						
3 µM 10 µM		No effect ↓ (87%)	No effect ↓ (49%)	No effect ↓ (79%)	n.s. n.s.	n.s. n.s.	n.s. n.s.
DHβE (10 µM), Supplemental Fig. S6*A*	Neuronal nAChR competitive antagonist; preference for β2-subtypes	No effect	No effect	No effect	n.s.	n.s.	n.s.
MLA (1 µM), Supplemental Fig. S6*B*	Neuronal nAChR competitive antagonist, selective for α7 subunit	No effect	No effect	No effect	n.s.	n.s.	n.s.
Atracurium (5 µM), Fig. 4*A*	Neuromuscular nAChR antagonist	↓ (85%)	↓ (55%)	↓ (72%)	n.s.	n.s.	n.s.
Tubocurarine, Fig. 4, *B–D*	Potent muscle nAChR antagonist at nM con						
0.1 µM 1 µM10 µM	centrations; neuronal nAChR antagonist at µM concentrations	No effect ↓ (95%) ↓ (93%)	No effect ↓ (80%) ↓ (83%)	No effect ↓ (79%) ↓ (94%)	n.s. n.s. n.s.	n.s. n.s. n.s.	n.s. n.s. n.s.
Atropine (1 µM), Fig. 5	Muscarinic receptor antagonist (competitive)	↓ (92%)	↓ (74%)	↓ (87%)	n.s.	n.s.	n.s.
α,β-mATP (10 µM), Fig. 5	Purinergic receptor desensitizer	No effect	No effect	No effect	n.s.	n.s.	n.s.
Effect of epibatidine (10 µM) on bethanechol (30 µM)-induced contraction (%KCl), Supplemental Fig. S9	Bethanechol is a selective muscarinic receptor agonist	No effect	No effect	No effect	n.s.	n.s.	n.s.

↑, activation of contraction; ↓, inhibition of contraction; α,β-mATP, α,β-methylene adenosine triphosphate; DHβE, dihydro-β-erythroidine hydrobromide; DMPP, 1,1-dimethyl-4-phenyl-piperazinium iodide); KCl, potassium chloride; KCl-induced contractions presented as medians (with their interquartile ranges); MLA, methyllycaconitine; Na^+^, sodium; nAChR, nicotinic acetylcholine receptor; n.s., nonsignificant; ObNT-Reinn, obturator nerve transfer reinnervated; TTX, tetrodotoxin. *P < 0.05.

### Voltage-Gated Sodium Channel Blocker Tetrodotoxin, but Not A-803467, Has Differential Effects on Epibatidine-Induced Strip Contractions

**Figure 1.**
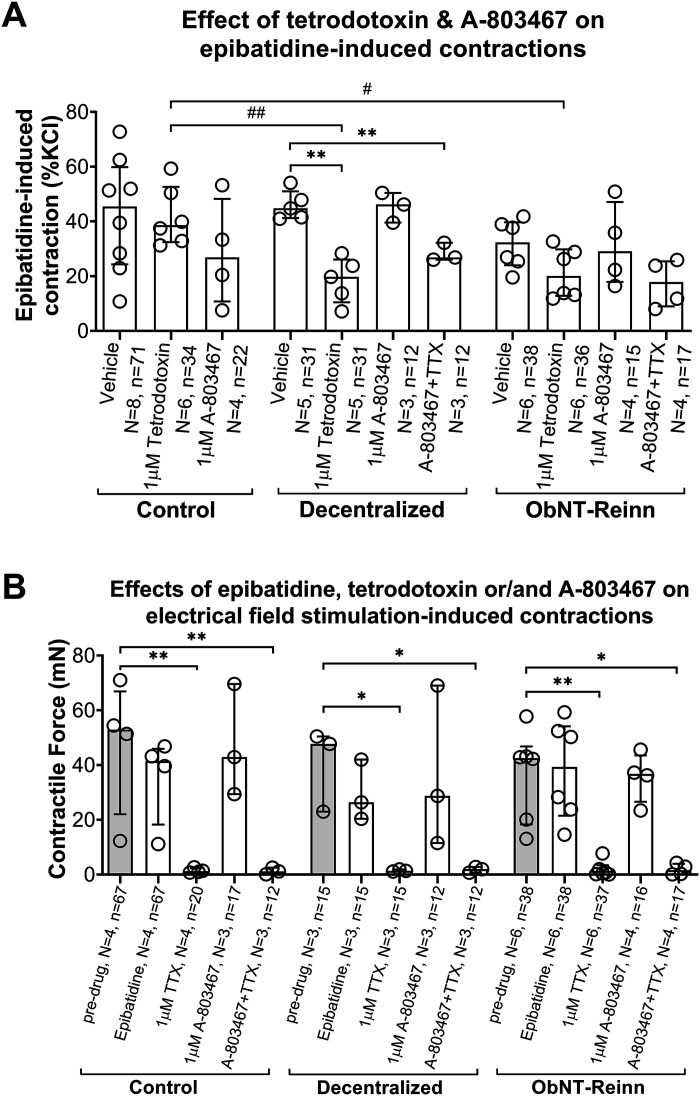
Effect of sodium channel blockade on epibatidine-induced strip contractions. *A*: responses to 10 µM epibatidine after treatment with vehicle, 1 µM of the sodium channel blocker tetrodotoxin (TTX), or 1 µM of the tetrodotoxin-resistant Na_v_1.8 sodium channel specific antagonist A-803467, in strips from control (male and female), Decentralized (female), and obturator-to-pelvic nerve transfer reinnervated (ObNT-Reinn; female) bladders, or 1 µM TTX plus 1 µM A-803467 in strips from Decentralized (female) and ObNT-Reinn (female) bladders. Responses are expressed as percentage of potassium chloride (KCl) response. *B*: the effect of drug treatments (listed in *A*) on electrical field stimulation (EFS)-induced strip contractions in control (male and female), Decentralized (female), and ObNT-Reinn (female) bladders and compared with predrug responses to EFS. *N* = number of bladders, and *n* = number of strips per group, with specifics indicated in the figure. Data are presented as medians (with their interquartile ranges, IQR). *P < 0.05 and **P < 0.01 compared with vehicle treatment in *A* and compared with predrug response in *B*, #*P* < 0.05 and ##*P* < 0.01 compared with the matching treated control group.

To investigate whether epibatidine-induced neurotransmitter release and subsequent strip contractions involved the generation of neuronal action potentials, muscle strips were incubated with 1 µM of tetrodotoxin (TTX), a voltage-gated sodium channel blocker that inhibits axonal action potential firing in neurons (Fig. 1*A*, Table 1). Pretreatment with 1 µM TTX had no effect on epibatidine-induced contractions in strips of control bladders, reported as medians (IQR): 52% (37–65) versus 39% (32–53). Yet, in Decentralized bladders, pretreatment with 1 µM TTX inhibited ∼56% of epibatidine-induced contractions compared with vehicle treatment, as medians [IQR: 45% (41–51) vs. 20% (10–26), *P* = 0.002, Fig. 1*A*]. In ObNT-Reinn bladders, pretreatment with 1 µM TTX had no statistically significant effect on epibatidine-induced strip contractions compared with vehicle [∼36% inhibition, as medians (IQR): 32% (24–40) vs. 20% (13–30)]. However, the response to pretreatment with 1 µM TTX before epibatidine treatment was significantly lower by ∼49% and 47% in Decentralized and ObNT-Reinn bladders, respectively, compared with TTX treatment in control bladders (*P* < 0.01 and 0.02, respectively, Fig. 1*A*, Table 1).

The effect of a potent and highly selective TTX-resistant sodium channel Na_v_1.8-specific blocker, A-803467 (1 µM, Table 1), on epibatidine-induced contractions was examined to determine whether TTX-resistant sodium channels contributed to action potentials. A-803467 did not block epibatidine-induced contractions in control bladder strips, compared with vehicle (Fig. 1*A*, Table 1). Similarly, A-803467 did not block epibatidine-induced contractions in Decentralized or ObNT-Reinn bladder strips (Fig. 1*A*, Table 1). To further confirm the effect of TTX in Decentralized and ObNT-Reinn bladders, strips from these animals were pretreated with TTX and A-803467 in combination. This combined treatment inhibited ∼40% of epibatidine-induced contractions, in Decentralized bladders, as medians (IQR): [45% (41–48) vs. 27% (26–32), *P* = 0.01, Fig. 1*A*], and ∼45% of epibatidine-induced contractions in ObNT-Reinn bladders [38% (30–41) vs. 18% (9–25), Fig. 1*A*]. The latter finding was not statistically significant, likely due to the variability in ObNT-Reinn vehicle and A-803467 + TTX treatment data.

We also examined the effects of TTX, A-8034367, and TTX plus A-803467 pretreatments, followed by epibatidine treatment, on EFS-induced strip contractions (Fig. 1*B*). TTX treatment followed by epibatidine treatment inhibited ∼97%, 95%, and 98% of strip response to EFS in control, Decentralized, and ObNT-Reinn bladders, respectively, compared with predrug results. In contrast, A-803467 treatment had no effect on strip responses to EFS in any group. The combined TTX and A-803467 treatments inhibited ∼97%, 92%, and 96% in the control, Decentralized, and ObNT Reinn, respectively. These results indicate that voltage-gated sodium channels that are sensitive to TTX are located on the prejunctional nerve fibers and TTX-resistant sodium channels are not involved in nerve-evoked canine bladder contractile response.

### Ganglionic Neuronal Nicotinic Receptor Antagonists Hexamethonium and Mecamylamine Inhibit Epibatidine-Induced Strip Contractions in the Three Surgical Groups

**Figure 2.**
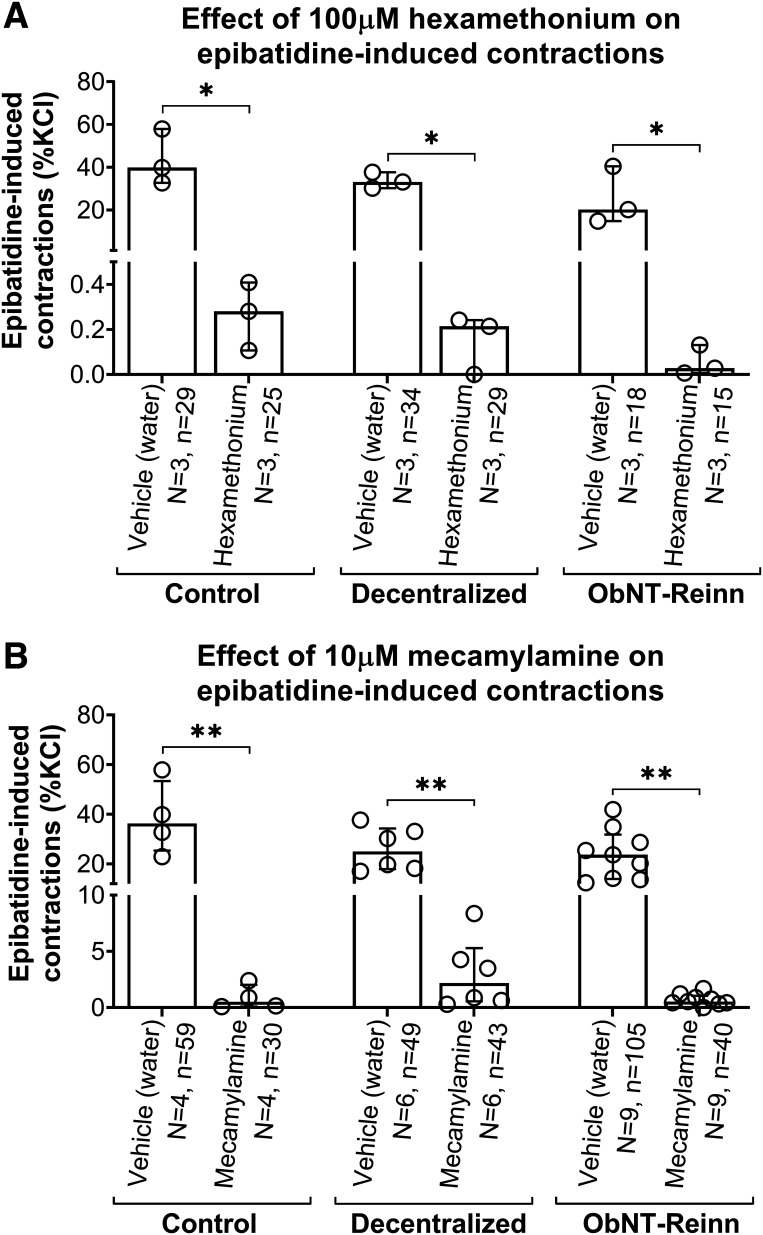
Effect of ganglionic nicotinic receptor antagonism on epibatidine-induced strip contractions. *A*: responses to 10 µM epibatidine after treatment with vehicle or 100 µM of the ganglionic antagonist hexamethonium in bladder strips from each group. *B*: responses to 10 µM epibatidine after treatment with vehicle or 10 µM ganglionic antagonist mecamylamine. Responses are expressed as percentage of potassium chloride (KCl) response. *N* = number of bladders, and *n* = number of strips per group, with specifics indicated in the figure. Data are presented as medians (interquartile ranges, IQR). *P < 0.05 and **P < 0.01 compared with vehicle treatment.

To determine if ganglionic (neuronal) nAChRs were taking part in the epibatidine-induced activity, the ganglionic nAChR antagonist, hexamethonium (Table 1), was first used. Hexamethonium is a primarily ganglionic blocker that blocks synaptic transmission in autonomic ganglia by a nondepolarizing postsynaptic action (37, 38). Hexamethonium (100 µM) suppressed ∼99%–100% of epibatidine-induced contractile activity in strips from control, Decentralized, and ObNT-Reinn bladders (Fig. 2*A*, Table 1).

We also examined the effects of the noncompetitive ganglionic nAChR antagonist mecamylamine on epibatidine-induced strip contractions (Table 1). Mecamylamine is primarily a nicotinic parasympathetic ganglionic blocker. Mecamylamine (10 µM) suppressed ∼97%, 87%, and 97% of epibatidine-induced activity in strips from control, Decentralized, and ObNT-Reinn bladders compared with vehicle treatments (Fig. 2*B*, Table 1).

We also examined the effects of a nonselective nAChR agonist, DMPP, that acts as a ganglion-stimulating agent. DMPP (100 µM) induced only small contractions in each group (Supplemental Fig. S5, Table 1). These contractions were ∼5%–7% of KCl-induced contractions and ∼17%–23% of epibatidine-induced contractions for all groups.

Taken together, results indicate that nAChRs involved in epibatidine-induced contractions of dog bladder are ganglionic subtypes.

### Subunit Selective Neuronal Nicotinic Receptor Antagonists SR 16584, DHβE, and Methyllycaconitine Have Differential Effects on Epibatidine-Induced Strip Contractions

**Figure 3.**
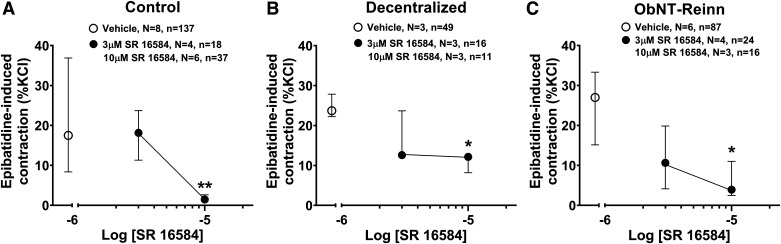
Effect of antagonism of neuronal (α3β4) receptor subunits on epibatidine-induced contractions. Responses to 10 µM epibatidine before and after treatment with vehicle (open circles) or treatment with the neuronal (α3β4) selective antagonist SR 16584 at 3 and 10 µM (closed circles) for strips from control (male and female) (*A*), Decentralized (female) (*B*), and obturator-to-pelvic nerve transfer reinnervated (ObNT-Reinn, female) (*C*) bladders. Responses are expressed as percentage of KCl response. Data for each treatment was compared with its vehicle results. *N* = number of bladders, and *n* = number of strips per group, with specifics indicated in the figure. Data are presented as medians (IQR). *P < 0.05 and **P < 0.01 compared with vehicle treatment.

Since epibatidine is a potent, nonsubtype selective, nAChR agonist (39), we examined for nAChR subtypes involved in epibatidine-induced contractions in controls, and whether there were changes in nAChR subtypes after bladder decentralization with or without reinnervation. First, the neuronal (α3β4)-selective antagonist SR 16584 was used and the concentration-response inhibition curve of SR 16584 was evaluated in controls (Supplemental Fig. S1*B*). In control bladders, although treatment with SR 16584 at the low concentration of 3 µM had no effect on epibatidine activity (Fig. 3*A*, Table 1), a higher concentration of 10 µM inhibited ∼87% of epibatidine-induced contractions, compared with vehicle (*P* = 0.004). Similarly, in Decentralized and ObNT-Reinn bladders, 3 µM SR 16584 had no effect on epibatidine contractions (Fig. 3, *B* and *C*; Table 1), yet the 10 µM SR 16584 inhibited ∼49% and 79% of epibatidine-induced contractions, respectively (*P* = 0.05 and *P* = 0.03, respectively, Fig. 3, *B* and *C*). In contrast, two other nAChR subtype-selective antagonists, dihydroxy β-erythroidine (DHβE, selective for receptors with the β2 subunit including the α4β2 nAChR subtype) and methyllycaconitine (MLA, an α7 subunit homomer-specific antagonist), at the selected concentrations used, did not inhibit epibatidine-induced strip contractions in bladders of any group (Supplemental Fig. S6, *A* and *B*, Table 1).

Combined, these results suggest that the α3β4 complex is likely the main combination of neuronal nAChR subunits in dog intramural ganglion neurons.

### Neuromuscular Nicotinic Receptor Antagonists Atracurium and Tubocurarine Inhibit Epibatidine-Induced Strip Contractions

**Figure 4.**
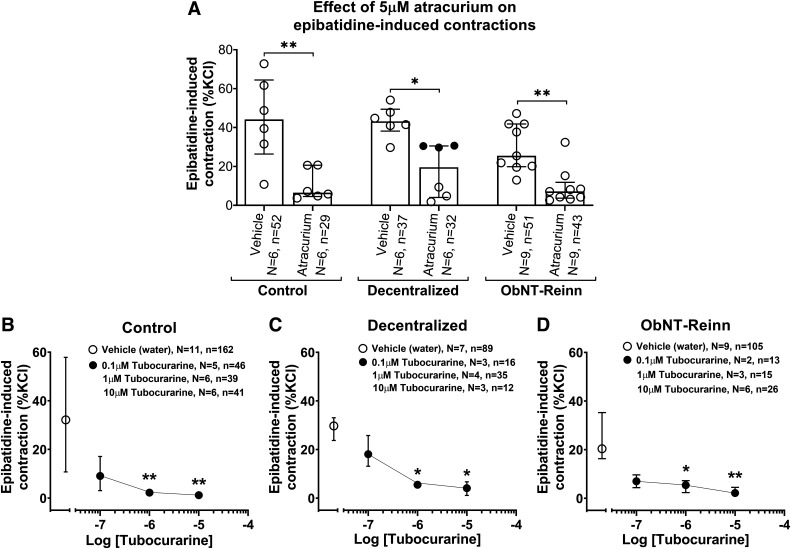
Effect of neuromuscular nicotinic receptor antagonism on epibatidine-induced strip contractions. *A*: responses to 10 µM epibatidine after treatment with vehicle or 5 µM neuromuscular nicotinic receptor antagonist atracurium in strips from control (male and female), Decentralized (female), and obturator-to-pelvic nerve transfer reinnervated (ObNT-Reinn, female) bladders. *B*–*D*: responses to 10 µM epibatidine before and after treatment with vehicle (open circles) or treatment with the neuromuscular nicotinic receptor antagonist tubocurarine at 0.1 µM, 1 µM, or 10 µM (closed circles) in strips from control (*B*), Decentralized (*C*), and ObNT-Reinn (*D*) bladders. Responses are expressed as percentages of KCl response. Data for each treatment was compared with its vehicle results. *N* = number of bladders, and *n* = number of strips per group, with specifics indicated in the figure. Data in *A*–*D* are presented as medians (interquartile ranges, IQR). When no error bars are seen, they are smaller than the symbols. *P < 0.05 and **P < 0.01 compared with vehicle treatment.

Treatment with the neuromuscular nAChR antagonist atracurium (5 µM) inhibited ∼85% of epibatidine-induced contractions in control bladder strips (*P* = 0.004, Fig. 4*A*, Table 1). Responses were 44% (26–64) versus 6% (4–21). In Decentralized and ObNT-Reinn bladder strips, atracurium inhibited ∼55% and 72% of epibatidine contractions, respectively (*P* = 0.01 and *P* = 0.008, respectively; Fig. 4*A*, Table 1). Responses were 43% (38–49) versus 19% (4–30) and 25% (20–42) versus 7% (4–12), respectively. Although the inhibition was slightly higher in control bladders, compared with Decentralized and ObNT-Reinn bladders, the response to preincubation of muscle strips with 5 µM atracurium before epibatidine treatment was similar between the three groups (Fig. 4*A*, Table 1).

Interestingly, in Decentralized bladders, atracurium inhibition was more prominent (∼88% inhibition) in the three dogs with shorter survival times postdecentralization (6–11 mo, open circles in Fig. 4*A*), yet less (∼32% inhibition) in the three dogs with longer survival times postdecentralization (18–21 mo, black circles in Fig. 4*A*). See Supplemental Fig. S7.

We also examined the effects of another neuromuscular nAChR antagonist, tubocurarine, on epibatidine-induced contractions by evaluating concentration-response inhibition curves (Supplemental Fig. S1*C*). In control bladder strips, treatment with tubocurarine at a lower concentration (0.1 µM) did not statistically significantly inhibit epibatidine-induced contractions (Fig. 4*B*). At higher concentrations of 1 µM and 10 µM, tubocurarine greatly inhibited epibatidine-induced contractions (∼95% and 93%, respectively; Fig. 4*B*, Table 1). In Decentralized and ObNT-Reinn bladder strips, again 0.1 µM tubocurarine did not inhibit epibatidine-induced contractions (Fig. 4, *C* and *D*). Similar to controls, at 1 µM or 10 µM, tubocurarine greatly inhibited epibatidine-induced contractions in Decentralized and ObNT-Reinn bladder strips (Fig. 4, *C* and *D*, Table 1), and the inhibition was ∼80% and 79% for 1 µM tubocurarine and ∼83% and 94% for 10 µM tubocurarine, respectively.

Since most of the commonly used drugs have off-target effects outside of nAChRs, e.g., potassium channels, we further tested the effects of atracurium and tubocurarine on bethanechol-induced contractions. At the utilized concentrations, neither of the two drugs had any effect on bethanechol-induced strip contractions in Decentralized and ObNT-Reinn bladders (Supplemental Fig. S8), This indicates that they are not likely having off-target effects.

### Muscarinic Receptor Antagonist Atropine Inhibits Epibatidine-Induced Strip Contractions

**Figure 5.**
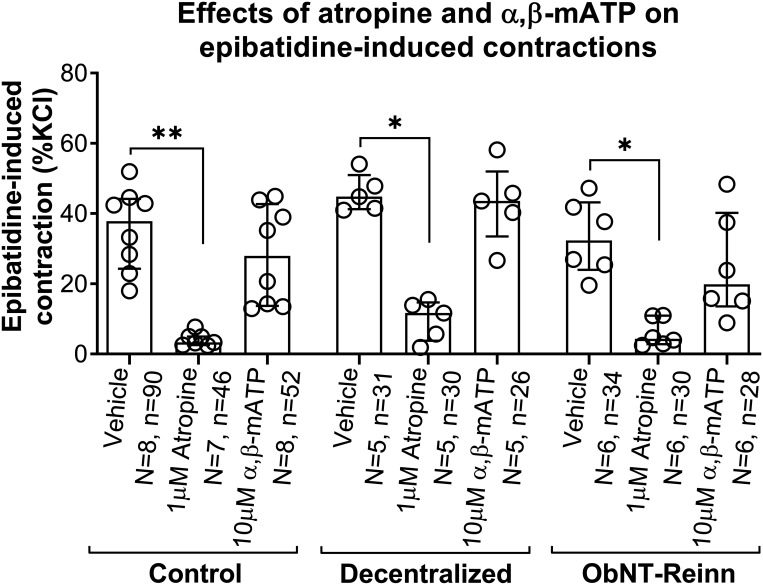
Effect of muscarinic and purinergic receptor antagonism on epibatidine-induced strip contractions. Responses to 10 µM epibatidine after treatment with vehicle (water), 1 µM of the muscarinic acetylcholine receptor (mAChR) antagonist atropine, or 10 µM of the purinergic receptor antagonist α,β-mATP, in strips from control (male and female), Decentralized (female), and obturator-to-pelvic nerve transfer reinnervated (ObNT-Reinn, female) bladders. Responses are expressed as percentage of the KCl response. *N* = number of bladders, and *n* = number of strips per group, with specifics indicated in the figure. Data are presented as medians (interquartile ranges, IQR). *P < 0.05 and **P < 0.01 compared with vehicle treatment.

We investigated whether mAChRs are involved in epibatidine-induced contractions. Preincubation of muscle strips with 1 µM of atropine, a nonsubtype selective competitive mAChR antagonist (Table 1), similarly inhibited epibatidine-induced contractions in control (∼92% inhibition, *P* = 0.003), Decentralized (∼74% inhibition, *P* = 0.02), and ObNT-Reinn (∼87% inhibition, *P* = 0.05), Fig. 5, Table 1).

### Epibatidine Does Not Block Bethanechol-Induced Strip Contractions

To prove that epibatidine-induced muscle strip activity was not due to the direct interaction of epibatidine with mAChRs and to further confirm the selectivity of epibatidine as nAChR agonist, we tested the effect of preincubating strips with 10 µM epibatidine before treatment with the mAChR agonist bethanechol (30 µM). Epibatidine pretreatment did not block or enhance bethanechol-induced strip contractions in the three groups compared with vehicle treatments (Supplemental Fig. S9). Yet, prior treatment with atropine completely blocked bethanechol-induced contractions in strips from all groups.

### Purinergic Receptor Antagonist α,β-mATP Does Not Inhibit Epibatidine-Induced Strip Contractions

To investigate whether purinergic receptors are also involved in epibatidine-induced activity, the bladder strips were incubated with a 10 µM of the nonsubtype selective purinergic receptor activator and desensitizer, α,β-methylene adenosine triphosphate (α,β-mATP). Desensitization of purinergic receptors with 10 μM α,β-mATP had no statistically significant effect on epibatidine-induced contractions in strips of any group compared with their vehicle treatments (the inhibition was only ∼21% in control, 6% in Decentralized, and 30% in ObNT-Reinn bladders, Fig. 5, Table 1). Also, the strips’ direct responses to 10 μM α,β-mATP treatment were similar across the groups (Supplemental Fig. S10).

## DISCUSSION

**Figure 6.**
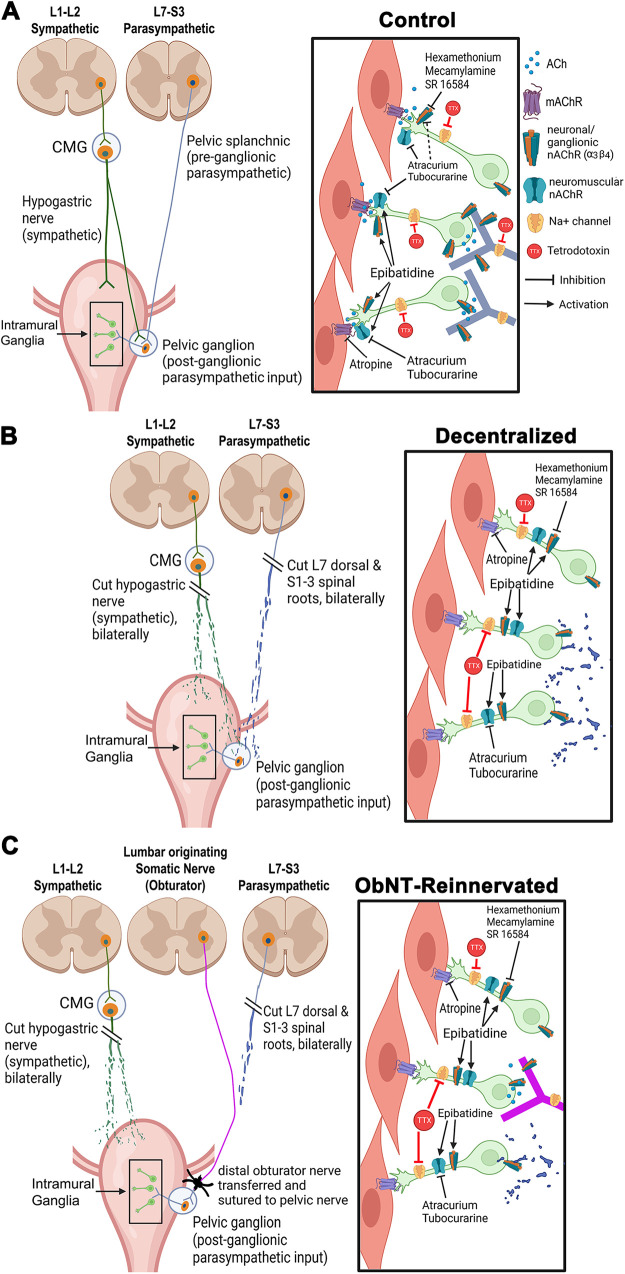
Summary diagram of the sympathetic and parasympathetic input to the bladder wall, distribution of nicotinic acetylcholine (ACh) receptor (nAChR) subtypes, and responses to nAChR agonist and antagonist treatments in bladder muscle strips from control (*A*), Decentralized (*B*), and Obturator nerve transfer reinnervated (ObNT-Reinn; *C*) animals. *A*, *left*: sympathetic innervation originates from preganglionic neurons in lumbar (L)1-L2 spinal cord segments. Their axons synapse in the caudal mesenteric ganglia (CMG) and then emanate to the bladder wall via the hypogastric nerve (which also sends axons to the pelvic ganglion). Parasympathetic innervation originates from preganglionic neurons in the L7-sacral (S)3 spinal cord segments. Their axons synapse in the pelvic ganglion on postganglionic neurons whose axons synapse on neurons in intramural ganglia in the bladder. *Inset*: enlargement of an intramural ganglion. In that ganglion, postganglionic parasympathetic axonal terminals from pelvic ganglia [neuronal (ganglionic) nAChR subtype] synapse on the intramural neuronal cell bodies. The intramural neurons are also of neuronal (ganglionic) nAChR subtype, with neuromuscular nAChR subtype on their terminals. Na^+^ channels shown on axons. Activation of nAChR on nerve terminals by epibatidine (agonist), releases the neurotransmitter ACh, which stimulates muscarinic ACh receptors (mAChRs) on the bladder smooth muscle causing contraction. Antagonism of *1*) prejunctional neuronal (α3β4) nAChRs by hexamethonium, mecamylamine, or SR 16548; *2*) neuromuscular nAChRs by atracurium or tubocurarine; or *3*) mAChR by atropine, inhibit epibatidine-induced contractions. However, blockade of Na^+^ channels on axons by tetrodotoxin (TTX) did not inhibit epibatidine induced activity. *B*, *left:* bladder was decentralized by bilateral transection of all sacral roots, dorsal roots of L7, and hypogastric nerves. *Inset*: relocation of prejunctional neuronal (α3β4) nAChRs from intramural ganglion nerve terminals to their axons, making their activity dependent on action potentials, supported by our findings that Na^+^ channel blockade by TTX inhibited epibatidine induced activity in these bladder strips. The neuromuscular nAChRs location and antagonism remained like controls. *C*, *left*: reinnervation surgery of the bladder was performed by the transfer and suture of obturator nerve to pelvic nerve. *Inset*: neuropathological changes in nAChR function or location associated with bladder decentralization were not ameliorated after the ObNT-Reinn surgery. Created with BioRender.com.

The nAChR agonist epibatidine is known to induce detrusor muscle contraction predominantly via the release of the neurotransmitter ACh from intramural nerve endings (4, 38). However, very little is known about the physiological characteristics of nAChRs in dog bladders. Therefore, utilizing in vitro pharmacological methods, we sought here to evaluate the responses of various nAChR subtypes that mediate bladder function in dogs with selective receptor agonists and antagonists. Our results are summarized in Table 1 and Fig. 6.

Focusing first on results from control bladders, we found that epibatidine-induced muscle strip contraction by mAChR activation (shown by the inhibition after atropine treatment), and via neuromuscular and neuronal α3β4 nAChR subtypes (Fig. 6*A*). Purinergic receptor activation was ruled out, as was TTX-resistant sodium channel activation. Sex differences were not evident in control strip responses to KCl or epibatidine (Supplemental Fig. S2, *A* and *B*). This is consistent with contractile responses to KCl observed in male and female rat and human bladder samples (40, 41).

TTX directly inhibits action potentials by blocking voltage-dependent sodium channels. Despite this sodium channel blockade, the continued epibatidine-induced contractions after 1 µM TTX treatment in control bladder strips (Fig. 1 and Fig. 6*A*) indicate that their epibatidine-induced activity is not mediated by epibatidine inducing the generation of action potentials, matching prior reports in bladders from other species (12, 15). TTX effectively blocked EFS-induced strip contractions (Fig. 1*B*) and ACh release, as previously reported (19), further indicating that EFS-induced strip contractions do not involve stimulation of nAChRs in the control bladders and suggest that the TTX-sensitive sodium channels responsible for the generation of action potentials are present on prejunctional axons. We speculate that these prejunctional nAChRs involved in neurotransmitter release and contractile responses are located near the transmitter release sites (i.e., on nerve terminals), as previously reported (38, 42–44). TTX-resistant sodium channels did not contribute to the generation of action potentials in control bladder strips, as shown by their lack of responsiveness to the potent and highly selective tetrodotoxin-resistant Na_v_1.8 sodium channel blocker A-803467 (Fig. 1). Thus, it is unlikely that tetrodotoxin-resistant Na_v_1.8 sodium channels are present on the axons of intramural nerves in dog bladder muscle.

The inhibition of epibatidine-induced strip contractions by hexamethonium and mecamylamine in control bladders (Fig. 2, *A* and *B* and Fig. 6*A*) indicates that neuronal ganglionic nAChRs are involved in the epibatidine-induced responses. Hexamethonium is an antagonist for both ganglionic (45) and neuronal nAChRs (46, 47), with relative specificity for the α3β4 subtype and receptors containing the α3 subunit (37, 48). Similarly, mecamylamine has high affinity for nAChR subtypes, particularly those with the α3 subunit (30), and it is more selective for β4- than β2-containing receptors (49). Although a ganglionic nAChR made up of α3 and β4 subunits is the most prevalent receptor subtype associated with bladder function (7), mecamylamine inhibits both neuromuscular and neuronal nAChRs (50, 51). However, mecamylamine is more selective for the neuronal type (it induces sustained inhibition of neuronal receptors, compared with its transient inhibition of neuromuscular receptors; 30). On the other hand, hexamethonium has weak competitive antagonism of neuromuscular nAChRs (45, 52, 53), and appears more competitive with ACh at autonomic ganglia than mecamylamine when applied in higher concentrations of ≥100 µM (45, 54, 55).

Therefore, nAChR subtypes involved in mediating bladder function were further examined using several subunit selective antagonists. In control bladders, the decrease in epibatidine-induced strip contractions after treatment with higher concentrations of the neuronal nAChR antagonist SR 16584 (Fig. 3*A*) strongly suggests that the α3β4 nAChR subtype is mediating much of the epibatidine-induced contractions. Epibatidine possesses high affinity for α3-containing ganglionic neuronal receptors, but relatively lower affinity for α1-containing receptors, e.g., the peripheral neuromuscular type α1β1δγ (53, 56). However, epibatidine is an indiscriminate nAChR agonist that acts on all nAChR subtypes (7, 39). Therefore, we used DHβE (a β2-containing nAChR selective antagonist) and MLA (a homomeric α7 selective antagonist). Neither had any effect on the epibatidine-induced muscle strip contractions in control bladders (Supplemental Fig. S6, *A* and *B*). DHβE is very selective for α4-containing receptors and has 100 times greater affinity for α4β2 than α3β4 (50), whereas epibatidine has greater affinity for α3 than α7 (57). Thus, it is reasonable to propose that most observed contractions occur as a result of activation of nAChRs without β2 or α7 subunits (58), and instead with α3β4 nicotinic subunits. We also tested the effect of the α3β4 selective antagonist, α-conotoxin AuBI on epibatidine-induced contractions in strips from one dog and three human bladders (with only small numbers of strips per bladder and per drug concentration) and found that at 1 µM of the toxin had no effect on epibatidine-induced contractions. However, at a higher concentration of 10 µM, it inhibited ∼75% of epibatidine-induced contractions (Supplemental Fig. S11). Thus, antagonists that are purely selective for a specific subunit containing subtype of nAChRs are largely not available. The reason behind the limited data on α-conotoxin AuBI is because this drug is very expensive, preventing extensive testing, and it easily loses its efficiency as an antagonist due to different factors, including poor production or handling during shipping that were beyond our control. However, despite this limitation, the α-conotoxin AuBI data strongly supports the involvement of the α3β4 nAChR subtype in mediating epibatidine-induced contractions.

Neuromuscular nAChR involvement was also investigated using two antagonists, atracurium and tubocurarine. The blockade of epibatidine-induced contractions by atracurium in control strips (Fig. 4*A* and Fig. 6*A*) could suggest that a neuromuscular nAChR contributes to the epibatidine excitatory effects, although this receptor type, specifically the adult type (α1)_2_β1δε, does not usually play a role in bladder function (59). It is worth noting that atracurium can also act on ganglionic nAChRs, e.g., the α3β4 nAChR, in the micromolar range (60). The inhibition of epibatidine contractions at the two higher concentrations of tubocurarine (1 and 10 µM) in control strips (Fig. 4*B*) is in agreement with prior reports that tubocurarine, a selective neuromuscular junction nAChR inhibitor (Table 1), blocks ACh-induced muscle membrane depolarization at the junction (61). Tubocurarine blocks neuronal nAChRs at a micromolar concentration, and blocks the neuromuscular type at a nanomolar concentrations (26). In alignment with our findings, α-conotoxin MII can selectively inhibit neuronal nAChR subtypes in nanomolar concentrations (62), although it can also inhibit other combinations of neuronal nAChR subunits and the neuromuscular type of nAChRs in micromolar concentrations (63, 64).

Finally, in control bladders, inhibition of epibatidine-induced muscle strip contractions by the mAChR antagonist, atropine (Fig. 5) indicates that the activity induced by epibatidine was dependent on mAChR activity and thus ACh release (Fig. 6*A*). This matches prior studies showing that epibatidine activates nAChRs on presynaptic nerve terminals to cause ACh release, which then acts on mAChRs located on muscle fibers, inducing contraction (4, 38). However, the lack of inhibition of bethanechol-induced contractions by epibatidine in control bladders (Supplemental Fig. S9) indicates that 10 µM epibatidine induces its effect by stimulating nAChRs without binding to mAChRs, as previously reported (23, 25, 65). The complete blockade of the maximally effective bethanechol concentration by atropine proves that the chosen concentration of atropine (1 µM) is sufficient to completely block the mAChR mechanism, without having antinicotinic effects. It is possible that when mAChRs on the detrusor smooth muscle are blocked by atropine, the effect of epibatidine in inducing muscle contraction would be diminished since the cholinergic response would be abolished even if the release of ACh was increased (12).

The lack of inhibition of epibatidine-induced contractions by the purinergic antagonist, α,β-mATP, in control bladder strips (Fig. 5) indicates that ACh dominates over ATP in transmitting epibatidine-induced strip activity in dog bladders (66). Since the urinary bladder receives different types of innervation in addition to the cholinergic innervation, we suggest that epibatidine-induced strip contractions are mainly mediated through the action on nAChRs and the release of ACh. However, we cannot exclude the notion that epibatidine could also stimulate the release of other mediators and their receptors taking part in mediating these contractions (15).

Combined, in control dog bladders, we propose that the effects of epibatidine are mostly driven by neuronal nAChR subtypes, particularly those with the α3β4 subunits, although there appears to be a partial contribution from the neuromuscular type nAChR (Fig. 6*A*). Hence, the multiplicity of nAChRs in the bladder and their specific characteristics should be considered when interpreting the differential effects of antagonists commonly used to block those receptors, especially since each receptor subtype possesses different pharmacological and physiological properties.

Our second objective was to examine whether the functionality of nAChRs and their responses to different receptor agonists and antagonists were altered after either long-term decentralization of the bladder, or long-term decentralization and then reinnervation, followed by a 12-mo recovery period. In each group, an extensive decentralization was needed to eliminate squat-and-void postures (i.e., transection of all sacral roots, hypogastric nerves, and the dorsal root of L7, partially shown in Fig. 6*B*; 21). The long-term decentralized animals that underwent obturator-to-pelvic nerve and sciatic-to-pudendal nerve transfer surgeries (i.e., ObNT-Reinn, partially shown in Fig. 6*C*), recovered both sensory and motor functions by 1 year after the reinnervation surgery (19, 21, 67, 68). We sought now to determine if there are long-term consequences of the decentralization and/or reinnervation on cholinergic nAChR subtypes or functionality.

The only difference observed from controls was the effect of TTX on epibatidine-induced contractions (Fig. 1*A* and Fig. 6, *B* and *C*; Table 1). Long-term bladder decentralization, with or without reinnervation, resulted in a reduction in epibatidine-induced bladder muscle strip contractions following treatment with the sodium channel blocker TTX, a reduction not seen in strips from control bladders (Fig. 6, *B* and *C*). We speculate that this is due to a neuropathological change in nAChR function or location as a result of the decentralization that was not ameliorated by the reinnervation. The average detrusor muscle strip responses to KCl, observed in Decentralized and ObNT-Reinn animals, were similar to controls (Supplemental Fig. S3), matching our prior report of no differences between average detrusor muscle strip responses to KCl in female unoperated or sham animals versus female Decentralized animals (19). These results also suggest a continued preservation of smooth muscle contractility after long-term decentralization, as previously reported for 12-mo Decentralized animals (20), likely due to the presence of intramural ganglia within the detrusor muscle wall. Nor were there differences in responses to epibatidine in bladder strips between the three groups (Supplemental Fig. S4), indicating that the overall net effects of nAChR activation on bladder function did not change after either procedure.

In contrast to controls, the inhibition of epibatidine-induced contractions by TTX in strips from Decentralized and ObNT-Reinn bladders (Fig. 1*A*) indicates that epibatidine-induced activity has become more dependent on its ability to generate action potentials, perhaps as a result of the long-term decentralization and associated neuropathology that remained even in the reinnervated bladders. Specifically, after nAChR activation by epibatidine, transmission of membrane depolarization to the transmitter release sites required activation of sodium channels in Decentralized and ObNT-Reinn bladders. We speculate that the decentralization disrupted the axons mediating bladder contraction, and that the nAChRs responsible for TTX-sensitive activity migrated along the axons to sites distant from the transmitter release sites, making their activity on ACh release dependent on action potentials, as diagramed in Fig. 6 and described previously (38, 69, 70). In addition, following axonal damage due to nerve transection, unusual chemosensitivity of the damaged axons might occur that involves changes in the receptors of damaged nerves (18, 71). Since, the effects of TTX and A-803467 on EFS-induced strip contraction in Decentralized and ObNT Reinn bladders were similar to that of controls, it is reasonable to speculate that the expression of sodium channels that contribute to neuronal excitability was not altered after nerve injury in this dog model, although prior rat studies indicated a plasticity in the expression levels of TTX-sensitive or TTX-resistant sodium channels following nerve injury (72–77).

Blockade of epibatidine-induced contractions by hexamethonium and mecamylamine observed in strips from Decentralized and ObNT-Reinn bladders were similar to control bladders (Figs. 2 and 6), demonstrating that the nAChR mediating the majority of the epibatidine responses in each group were the neuronal ganglionic receptor subtype. It is also similar to purinoceptors mediating ATP-induced contraction in rat gut walls in which a reduction of contractions was induced by hexamethonium (78).

The decrease in epibatidine-induced strip contractions after treatment with the higher concentration of the neuronal nAChR antagonist SR 16584 in the Decentralized and ObNT-Reinn bladders (Fig. 3, *B* and *C*), and the lack of effect after DHβE or MLA treatments, strongly suggests that the nAChR mediating epibatidine-induced contractions is the α3β4 subtype, similar to controls. The inhibition of epibatidine-induced strip contractions in the Decentralized and ObNT-Reinn bladders by atracurium, although to a lesser extent than that in strips from controls (Fig. 4*A*), suggests that neuromuscular type of nAChRs also contributes to epibatidine activity, as in controls.

### Perspectives and Significance

Collectively, the nature of the nAChRs mediating muscle contractions in these dog groups is somewhat unusual due to their sensitivity to antagonists thought to be selective for either neuromuscular junction or ganglionic nAChRs (79). In bladder smooth muscle from each group, nAChRs with α3β4 subunits, and possible neuromuscular nAChR types, appear to be the primary mediator(s) of epibatidine-induced contractions. One difference between the surgical groups was what appeared to be a relocation of nAChRs from the preneuromuscular junction to along the axon, after the extensive long-term bladder decentralization with or without reinnervation, rather than remaining at the axon terminal (as seen in the controls). This possible change in location of nAChR profile after long-term extensive decentralization suggests a physiological relevance for these receptors as a compensatory mechanism to recover bladder function and a potential target for drug treatment.

## DATA AVAILABILITY

Data will be made available upon reasonable request.

## SUPPLEMENTAL DATA

10.34944/dspace/8650 Supplemental Figs. S1–S11: http://dx.doi.org/10.34944/dspace/8650.

## GRANTS

This study was supported by the National Institute of Neurological Disorders and Stroke Grant R01NS070267 (to M.R.R. and M.F.B.). 

## DISCLOSURES

No conflicts of interest, financial or otherwise, are declared by the authors. 

## AUTHOR CONTRIBUTIONS

N.F., M.F.B., A.S.B., J.M.B., and M.R.R. conceived and designed research; N.F., M.F.B., D.G., D.S.P., A.S.B., E.T., A.A., J.M.B., B.R.J., S.F.B., and M.R.R. performed experiments; N.F., M.F.B., and D.G. analyzed data; N.F. interpreted results of experiments; N.F. prepared figures; N.F. drafted manuscript; N.F., M.F.B., B.R.J., and M.R.R. edited and revised manuscript; N.F., M.F.B., and M.R.R. approved final version of manuscript. 
